# Identifying key mechanisms leading to visual recognition errors for missed colorectal polyps using eye‐tracking technology

**DOI:** 10.1111/jgh.16127

**Published:** 2023-02-01

**Authors:** Omer F Ahmad, Evangelos Mazomenos, Francois Chadebecq, Rawen Kader, Mohamed Hussein, Rehan J Haidry, Juana González‐Bueno Puyal, Patrick Brandao, Daniel Toth, Peter Mountney, Ed Seward, Roser Vega, Danail Stoyanov, Laurence B Lovat

**Affiliations:** ^1^ Wellcome/EPSRC Centre for Interventional and Surgical Sciences University College London London UK; ^2^ Division of Surgery and Interventional Sciences University College London London UK; ^3^ Gastrointestinal Services University College London Hospital London UK; ^4^ Odin Vision Ltd London UK

**Keywords:** artificial intelligence, colonic polyps, colonoscopy, colorectal cancer

## Abstract

**Background and Aim:**

Lack of visual recognition of colorectal polyps may lead to interval cancers. The mechanisms contributing to perceptual variation, particularly for subtle and advanced colorectal neoplasia, have scarcely been investigated. We aimed to evaluate visual recognition errors and provide novel mechanistic insights.

**Methods:**

Eleven participants (seven trainees and four medical students) evaluated images from the UCL polyp perception dataset, containing 25 polyps, using eye‐tracking equipment. Gaze errors were defined as those where the lesion was not observed according to eye‐tracking technology. Cognitive errors occurred when lesions were observed but not recognized as polyps by participants. A video study was also performed including 39 subtle polyps, where polyp recognition performance was compared with a convolutional neural network.

**Results:**

Cognitive errors occurred more frequently than gaze errors overall (65.6%), with a significantly higher proportion in trainees (*P* = 0.0264). In the video validation, the convolutional neural network detected significantly more polyps than trainees and medical students, with per‐polyp sensitivities of 79.5%, 30.0%, and 15.4%, respectively.

**Conclusions:**

Cognitive errors were the most common reason for visual recognition errors. The impact of interventions such as artificial intelligence, particularly on different types of perceptual errors, needs further investigation including potential effects on learning curves. To facilitate future research, a publicly accessible visual perception colonoscopy polyp database was created.

## Introduction

Adenoma detection rate (ADR) is an independent predictor for the risk of interval colorectal cancer.^1^ Unfortunately, ADR remains highly variable amongst endoscopists. Lesions can be missed during colonoscopy due to inadequate mucosal exposure or failure to recognize lesions within the endoscopic field of view.

There is an appreciation that many lesions are endoscopically subtle and may be overlooked during colonoscopy. Indirect evidence supports this concept; for example, improved ADRs have been demonstrated in studies when nurses have acted as second observers, and more recently, artificial intelligence (AI)‐assisted polyp detection software use has been associated with higher ADRs when compared with conventional colonoscopy in randomized controlled trials.^2^, ^3^

There are limited studies offering mechanistic insights into the failed visual recognition of lesions. Preliminary observational data suggest that a learning curve may exist for the detection of flat and depressed colorectal lesions.^4^ Furthermore, eye‐tracking studies, which evaluate endoscopist visual gaze patterns, have been performed. Several studies have demonstrated differing viewing behaviors during normal colonoscopy withdrawals.^5^, ^6^ These studies did not, however, investigate visual gaze patterns in instances of missed lesions in the endoscopic field of view. More recent studies have compared visual gaze patterns on images of lesions using different imaging methods.^7^, ^8^ However, no eye‐tracking study to date has provided mechanistic insights into recognition errors.

In this study, we aimed to evaluate visual recognition errors for colonic lesions by using eye‐tracking equipment and to provide preliminary mechanistic insights alongside a comparison with an AI algorithm designed for subtle polyp detection.

## Methods

This was an observational study involving two phases.

The aim of phase 1 was to provide mechanistic insights into recognition errors for participants at the beginning or early phase of their training using still (static) images and eye‐tracking equipment. The aim of phase 2 was to validate these findings on videos.

### Phase 1: Combined eye‐tracking experiments and manual selection of polyp regions in endoscopic still images

#### Dataset

Thirty still images were extracted from colonoscopy video recordings in white light at high definition (1920 × 1080 resolution), using Olympus EVIS LUCERA CV290(SL) processors and colonoscopes. The procedures were performed by two expert (ADR > 50%) bowel cancer screening accredited colonoscopists (R. V. and E. S.).

The first five images in the experiments contained easy to identify polyps in the center of the image and clearly in view, which allowed participants to get accustomed to the study setup. The remaining 25 images in the study were presented in a random order, consisting of 20 images containing 25 polyps and 5 negative (non‐polyp) images to avoid operator bias. These polyp images were extracted from video sequences specifically at times where the polyps had only just been identified by the expert operator owing to subtle visual cues during “near miss” scenarios. This makes the dataset more relevant for real‐life clinical applications, as the polyps were not defined as being difficult to detect purely based on morphology or size, as the actual visual scenario encountered was responsible for detection difficulty. Three endoscopists (O. F. A., R. V., and E. S.) created ground truths for the polyp areas by placing a bounding box around the polyp region within the images, which was used only for evaluation purposes. All polyps were confirmed by histopathology. To facilitate future research, we created the publicly accessible UCL polyp perception dataset.

The polyp characteristics (size, morphology, location, and histopathology) are summarized in Table 1 for the UCL polyp perception database.

**Table 1:** Summary of polyp characteristics in the two datasets

UCL polyp perception database (still images)	UCL subtle video dataset
25 polyps	39 polyps
Mean size = 10.0 ± 7.3 mm	Mean size = 10.2 ± 7.3 mm
**Paris classification** ^†^	**Paris classification** ^‡^
Flat/flat elevated 72% (18)	Flat/flat elevated 69% (27)
Protruded 28% (7)	Protruded 31% (12)
**Location**	**Location**
Right 76% (19)	Right 77% (30)
Left 20% (5)	Left 18% (7)
Rectum 4% (1)	Rectum 5% (2)
**Pathology**	**Pathology**
LGD adenoma 52% (13)	LGD adenoma 46% (18)
SSL (no dysplasia) 44% (11)	SSL (no dysplasia) 54% (21)
SSL (LGD dysplasia) 4% (1) Advanced colorectal polyp 40% (10)	Advanced colorectal polyp 36% (14)

^†^ LST‐NG‐F (IIa) = 1; LST‐NG‐PD (IIa + IIc) = 1; LST‐G‐H (IIa) = 1.

^‡^ LST‐NG‐F (IIa) = 4; LST‐G‐H (IIa) = 1; LST‐G‐M (IIa + Is) = 1.

Advanced colorectal polyps are defined using the British Society of Gastroenterology post‐polypectomy surveillance guidelines.^16^ This includes adenomas > 10 mm, adenomas with high‐grade dysplasia, serrated polyp > 10 mm, and serrated polyp with dysplasia.

LGD, low‐grade dysplasia; SSL, sessile serrated lesion.

#### Experiment setup

A custom interface was designed for the experiments. A screen‐based Tobii X30‐120 eye‐tracker was attached to a 24‐inch monitor to record eye movements. The system uses near infrared light‐emitting diodes as light sources; corneal light reflections are captured by the cameras at 120‐Hz frequency. The hardware was calibrated prior to use in experiments for each participant. The eye‐tracker provided timestamped gaze locations in the form of pixel coordinates for each eye with the mean value ultimately considered the final gaze position. In post‐processing, gaze fixations were defined as consecutive gaze samples with an inter‐sample distance of less than 25 pixels (disregarding missing data) and with a minimal duration in milliseconds (50 ms); candidates below this duration threshold were disregarded.

**Figure 1:**
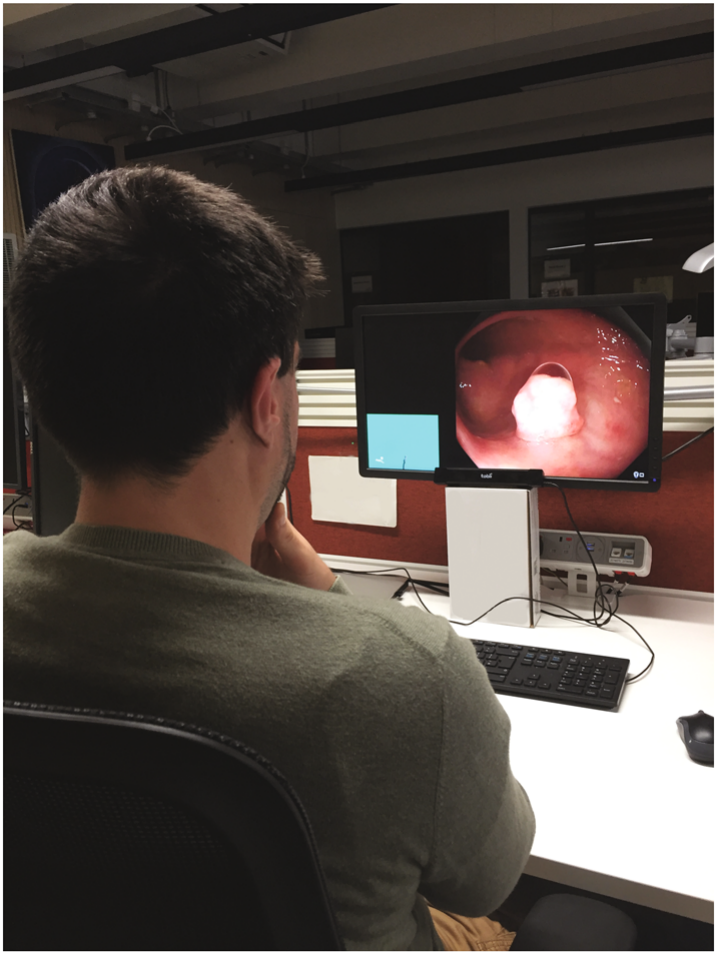
Eye‐tracking experiment setup. Screen‐based eye‐tracking device Tobii X30‐120 eye‐tracker was placed below a computer monitor to record eye movements.

The experiments were performed in a standardized manner in a non‐clinical setting using a 24‐inch monitor and observers maintained a fixed distance of approximately 65 cm away from the eye‐tracker as recommended by the manufacturer. The experiment setup is illustrated in Figure 1.

Participants were told that images may or may not contain polyps. Images were then displayed on a laptop with a 13‐inch monitor. Participants were instructed to initially observe each image for 12 s, as if they were searching for polyps during colonoscopy. Following this, a clear transition was made to an annotation phase of the experiment, where 10 s was provided to mark any areas suspicious for polyps with the cursor on the same image. This allowed for initial eye‐tracking data to be interpreted in the context of areas marked by the participants as suspicious for polyps. Images could only be viewed once.

Eye‐tracking experiments using videos were not appropriate for the aims of the study, as these would only produce cumulative gaze patterns over a large number of sequential video frames, not in relation to a fixed region such as a polyp, which would not provide clear mechanistic insights into reasons for failed lesion recognition.

### Phase 2: Video experiments

The same participants in phase 1 also took part in a video study to assess polyp detection ability. We used a perceptually challenging dataset entitled “the UCL subtle video dataset,” which consisted of 34 video clips containing 39 polyps in near miss scenarios. These were white light videos, collected by two expert colonoscopists (R. V. and E. S.); median video duration was 9.5 s (interquartile range 8.0–10.0). All polyps were confirmed by histopathology and the characteristics are summarized in Table 1. The videos were presented randomly and included seven negative videos (20% of all videos) in accordance with a similar published protocol to avoid operator bias.^9^

Custom software was created to allow viewing of videos once only. Participants were asked to pause the videos and place a bounding box around any area suspicious for a polyp on a frame. The participants were asked to pause the video only when they desired to mark the area that was deemed suspicious, to avoid the practice of pausing simply to allow for prolonged observation. Only a single marking was required for any structure that was suspicious for a polyp during each video sequence.

We compared the endoscopist performance in this study to a convolutional neural network (CNN) developed for polyp detection. Detailed descriptions of the CNN development and performance on the UCL subtle dataset have previously been published and are briefly summarized in the supporting information.^10^

### Participants

The study included 11 participants: 7 trainees (the Joint Advisory Group on Gastrointestinal Endoscopy non‐independent) and 4 medical students completely naïve to endoscopy. The aim was to specifically evaluate recognition errors at the start or early phase of training. These experiments were performed between December 2019 and October 2020.

### Outcomes

The primary outcome was per‐polyp sensitivity, defined as the total number of polyps correctly detected by a participant divided by the total number of polyps in the dataset. A correct detection was defined as one within the ground truth bounding box.

**Figure 2:**
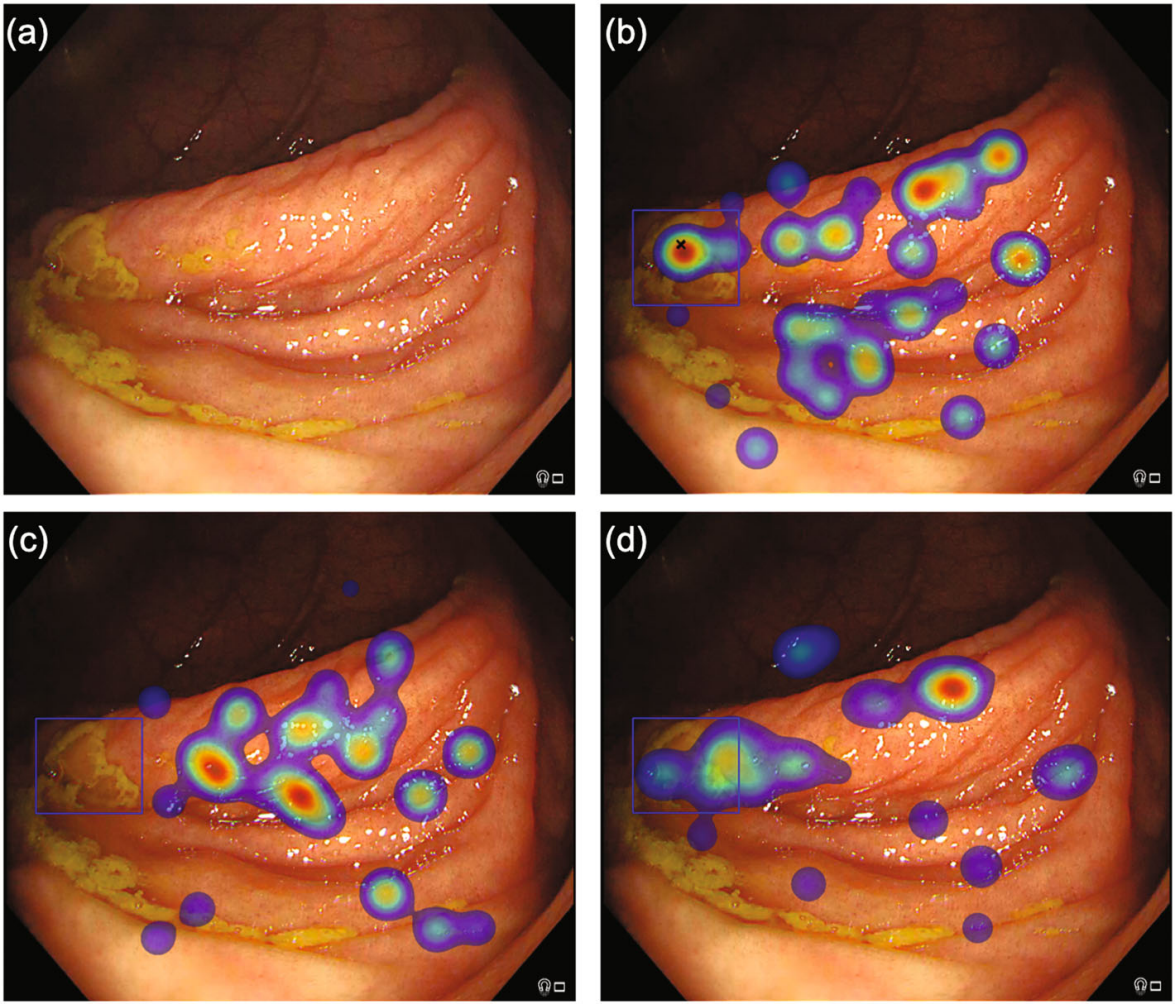
Eye‐tracking outputs for different participants viewing the same image. (a) Raw image with sessile serrated lesion highlighted in subsequent images with blue bounding box. (b) Correct detection with black cross indicating that the participant marked the polyp manually, corroborated with eye‐tracking heat map demonstrating fixations in the polyp region defined by the blue bounding box. The heat map colors represent the cumulative number of fixations, with red indicating a longer gaze (more fixations). (c) A gaze error is demonstrated; the participant did not mark the polyp manually (absence of cross) and also did not fixate on the region; that is, polyp was not observed. (d) A cognitive error is demonstrated; the participant did not mark the polyp manually (absence of cross) but fixated in the polyp region defined by the blue bounding box, suggesting that the polyp was observed.

The secondary outcomes included positive predictive values, definitions for the still images, and video experiments, which are provided in the supporting information. For the visual gaze experiments, recognition errors occurred whenever a polyp was not identified by the endoscopist in the annotation phase; that is, a mark was not placed in the bounding box corresponding to the polyp region. These recognition errors were then further categorized as “gaze errors” or “cognitive errors” based on eye‐tracking data. Gaze errors occurred when the participants did not fixate at all in the polyp region as defined by the bounding box; that is, the lesion was not observed or marked as a polyp. Cognitive errors occurred when the participants did fixate in the polyp region but did not annotate this; that is, the lesions were observed but not marked as suspicious. These definitions are illustrated in Figure 2.

### Statistical analysis

Parametric continuous variables are expressed as means with standard deviation and non‐parametric variables as medians with interquartile range. The Clopper–Pearson exact 95% confidence intervals (CIs) were calculated. Fisher's exact or *χ* ^2^ test was used to compare differences in categorical variables. *P* < 0.05 was defined as the threshold for statistical significance. GraphPad Prism (version 8) was used for statistical analyses.

### Ethics

The study was approved by the Cambridge Central Research Medical Ethics Committee (REC reference no. 18/EE/0148).

## Results

### Phase 1: Combined eye‐tracking experiments and manual selection of polyp regions in endoscopic still images

Trainees detected more polyps compared with medical students, with per‐polyp sensitivities of 49.1% (95% CI, 41.5–56.8%) and 35.0% (95% CI, 25.7–45.2%), respectively (*P* = 0.0283). Positive predictive values were also significantly higher for trainees who achieved 62.8% (95% CI, 54.1–71.0%) compared with 35.4% (95% CI, 26.0–45.6%) for medical students (*P* < 0.0001).

**Figure 3:**
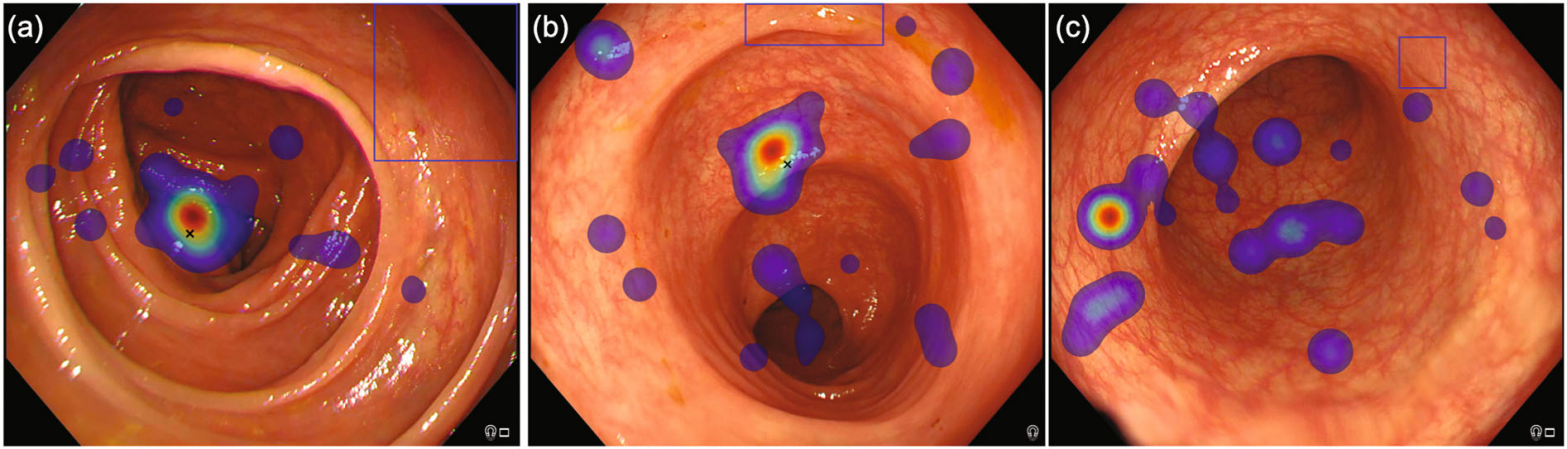
Gaze errors highlighted by the absence of both manual marking (black cross) and visual gaze in polyp region defined by the blue bounding box. (a) LST‐NG‐F subtype (Paris IIa, 30 mm). (b) Sessile serrated lesion (Paris Is, 9 mm). (c) Adenoma (Paris IIa, 6 mm).

**Figure 4:**
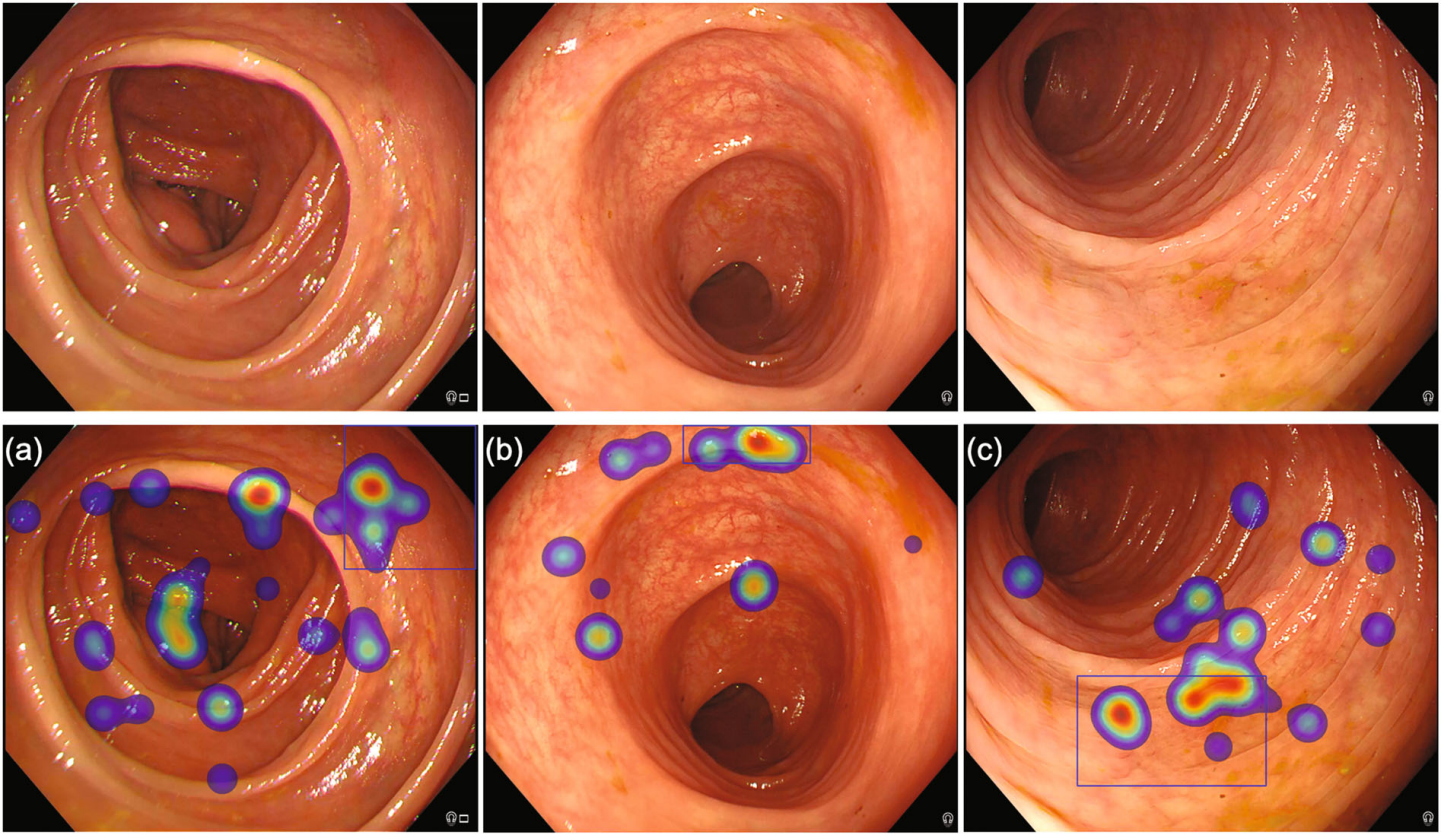
Cognitive errors highlighted by the absence of manual marking (black cross) combined with fixations within the polyp region as defined by the blue bounding box as illustrated by the heat maps (red indicating longer duration gaze, i.e. more fixations), indicating that the participant did observe polyp. (a) LST‐NG‐F subtype (Paris IIa, 30 mm). (b) Sessile serrated lesion (Paris Is, 9 mm). (c) Sessile serrated lesion (Paris IIa, 10 mm).

Overall, cognitive errors occurred more frequently than gaze errors, accounting for 65.6% (95% CI, 57.5–73.0%) and 34.4% (95% CI, 27.0–42.5%) of errors, respectively. Cognitive errors represented a significantly greater proportion of errors in trainees when compared with medical students, accounting for 73.0% (95% CI, 62.6–81.9%) and 55.4% (95% CI, 42.5–67.7%), respectively (*P* = 0.0264). The recognition errors are summarized in Table 2. The different types of perceptual errors are illustrated in Figures 3 and 4.

**Table 2:** Recognition errors categorized in trainees and medical students

	Gaze errors [95% CIs] (*n*)	Cognitive errors [95% CIs] (*n*)
Trainees	27.0% [18.1–37.4] (24/89)	73.0% [62.6–81.9] (65/89)
Medical students	44.6% [32.3–57.5] (29/65)	55.4% [42.5–67.7] (36/65)

CIs, confidence intervals.

### Phase 2: Video observation and comparison with convolutional neural network

The CNN detected significantly more polyps than trainees and medical students, with per‐polyp sensitivities of 79.5% (95% CI, 63.5–90.7%), 30.0% (95% CI, 24.7–35.9%), and 15.4% (95% CI, 10.1–22.0%), respectively (*P* < 0.0001).

The positive predictive values on a per‐polyp basis were 56.9% (95% CI, 48.4–65.2%) and 40.7% (95% CI, 28.1–54.3%) for trainees and medical students, respectively. It was not possible to report positive predictive values on a per‐polyp basis for the CNN as predictions were made on all frames; the model achieved a per‐frame specificity of 70.5% (95% CI, 69.3–71.8%).

## Discussion

Failure to recognize colorectal lesions present in the endoscopic field of view, particularly subtle and advanced neoplasia, has gained more attention recently, with increased availability of image‐enhanced endoscopy and AI‐assisted polyp detection software.^11^ Mechanistic insights into failed recognition are lacking. In this study, we demonstrated mechanistic differences in polyp recognition errors, with cognitive errors representing the major overall type of error and reason for missed lesions. Finally, we validated our findings on a perceptually challenging video dataset, enriched with subtle and advanced neoplasia, also demonstrating that an AI algorithm detected significantly more lesions than trainees and medical students.

There are limited studies that specifically mechanistically investigate standalone polyp recognition skills and inter‐observer variability in endoscopic image perception. Previously published eye‐tracking studies have evaluated cumulative visual gaze patterns during colonoscopy withdrawals, with one preliminary study suggesting an association between central gaze patterns and ADRs.^5^, ^6^ However, these focused on overall colonoscopy viewing behavior during normal withdrawals and did not address lesion recognition directly. Recently, Troya *et al*. evaluated the impact of AI‐based polyp detection software using eye‐tracking glasses, demonstrating that AI detected polyps faster than humans; however, it also increased misinterpretation of normal mucosa and decreased participant eye travel distance.^12^ Meining *et al*. demonstrated that viewing behavior differed when comparing paired narrow‐band imaging and white light images of endoscopic lesions, with more fixations and total time spent on narrow‐band imaging images.^8^ More recently, Kumahara *et al*. conducted an eye‐tracking study involving 10 endoscopists (1 expert and 9 non‐experts), comparing miss rates on matched still images of 30 polyps captured using white light, blue‐laser imaging, and linked‐color imaging.^7^ The reported miss rate for white light was 14.7% for the non‐experts, and no lesions were missed by the expert. The detection rates are much lower in our study, even though we allowed double the viewing time for still images (10 s). This could be due to our dataset containing extremely subtle lesions, including a high proportion of flat lesions and sessile serrated lesions, owing to our novel method of extracting still frames from video sequences when visual cues appeared in near miss scenarios. Also, crucially, our dataset contained a high proportion of advanced colorectal polyps, while the dataset used by Kumahara *et al*. almost exclusively contained diminutive polyps, making our findings highly relevant to interval colorectal cancer prevention. Moreover, our novel study design offered mechanistic insights by asking participants to mark suspicious areas for polyps, in combination with eye‐tracking data. Our study is the first to introduce the concepts of different types of perceptual errors, most notably cognitive errors. Previous studies have simply considered that an observation or fixation on a polyp region constitutes a detection, although our study demonstrates otherwise.

It is widely recognized that polyp recognition errors could represent an important contribution to the development of interval colorectal cancers.^13^ Also, the association with procedure volume suggests that a learning curve exists. Currently, polyp recognition skills are not formally taught or incorporated into endoscopic curricula, although previous quality improvement studies have included pattern recognition training for subtle visual cues.^14^ Future studies should investigate the impact of such interventions on perceptual skills. In addition, in our study, cognitive errors represented the major perceptual error type. Interventions are needed to specifically address this aspect rather than just gaze errors. It is possible that technologies that increase visibility alone, such as image‐enhanced endoscopy, may not adequately compensate for cognitive errors. Our study also demonstrated that at a CNN was able to detect more polyps than trainees. Further studies are required to evaluate the impact of AI overlays on trainees, particularly to determine if these would convert cognitive errors to true detections and establish whether AI could shorten the learning curve for polyp recognition.^15^ Ideally, studies evaluating the use of AI should include endoscopist–AI interaction; that is, videos should include CNN overlays, to determine impact on endoscopist polyp detection performance.

The limitations of this study include its conduct outside of a clinical setting, with participants making judgements while observing selected images. We attempted to mitigate this by also using video experiments, although for eye‐tracking experiments, videos were not feasible, as this would only produce cumulative gaze patterns over a large number of sequential video frames, not in relation to a fixed region, which would not provide clear mechanistic insights into reasons for failed polyp recognition. Additional visual gaze studies on videos could however provide benefit by investigating whether cumulative gaze patterns, such as in the periphery or center of the screen, could contribute to and correlate with gaze‐type errors as defined in this study. Further studies should also ideally evaluate perceptual skills during endoscopy, although this is challenging as the reference or gold standard for detected polyps will be determined by the performing endoscopist and will also depend on other factors such as mucosal exposure skills. Moreover, it would not be possible to study inter‐observer variability in a clinical environment. Finally, our eye‐tracking phase did not include expert participants, as we focused on trainees and naïve students given the lower detection rate in this group and to evaluate the learning curve for those at the beginning or early stage of endoscopy training. Future studies should also evaluate experts, to determine if perceptual errors differ in comparison with non‐experts. Further research can be facilitated with the availability of the publicly accessible database produced and the novel framework provided by this study.

In conclusion, our study provided a novel approach and definitions for perceptual errors related to polyp recognition. Eye‐tracking studies demonstrated that cognitive errors accounted for the majority of perceptual errors. Further efforts should investigate the impact of interventions, including educational initiatives and technological solutions such as AI, on specific types of perceptual errors in endoscopists.

## Supporting information


**Data S1.** Supporting Information.

## Data Availability

To obtain access to the UCL polyp perception database, researchers should email l.lovat@ucl.ac.uk. Upon agreeing the terms of use, an electronic weblink will be sent by email to download the images and associated meta‐data.
