# Supplementary material for: Identifying key mechanisms leading to visual recognition errors for missed colorectal polyps using eye‐tracking technology
Source: J Gastroenterol Hepatol. 2023 Feb 1;38(5):768–74. doi: 10.1111/jgh.16127 (PMC10601973; doi:10.1111/jgh.16127)
Supplement: Supplementary file 1 — Data S1. Supporting Information. [file JGH-38-768-s001.docx]

**Supplementary Material**

Algorithm development, including descriptions of the training dataset have been previously published in detail ^1^. The key aspects are summarised below.

**Algorithm development**

A fully-convolutional network (FCN) with a ResNet-101 backbone architecture was used. The model was trained with Pytorch on an NVIDIA GeForce RTX 2080 Ti GPU.

The training dataset for the algorithm consisted of two datasets (Dataset A and B). Dataset A was a video dataset which was created at University College London Hospitals, consisting of 33 complete colonoscopy withdrawals, collected using Olympus EVIS LUCERA CV290(SL) processors and colonoscopes. This included 53,849 polyp positive frames (158 polyps) and 5000 polyp negative frames. Only white light frames were used. Polyp positive frames were manually annotated with bounding box. Dataset B was created from public datasets, including CVC-ColonDB300, CVC-ClinicDB612, CVC-ClinicHDSegmentTrain and CVC-Video databases, consisting of 10,993 polyp positive frames which were a mixture of video and still images.

**Performance Metrics**

True positive (TP) = endoscopist manual prediction overlaps with the ground truth polyp bounding box.

False positive (FP) = endoscopist manual prediction on a non-polyp area i.e. not overlapping with the ground truth polyp bounding box

Per polyp sensitivity = number of polyps correctly detected / total number of polyps in dataset

Positive predictive value = TP/ (TP + FP)

Per-polyp positive predictive value (video experiments) = true positive polyp predictions on a per-polyp basis divided by total number of positive predictions made by endoscopists.

**References**

1. Ahmad OF, González-Bueno Puyal J, Brandao P, Kader R, Abbasi F, Hussein M, et al. Performance of artificial intelligence for detection of subtle and advanced colorectal neoplasia. Dig Endosc. 2022;34:862-869.
